# Occurrence data for habitat suitability modelling to support conservation planning in Latvia: species list, observations and ecogeographical variable selection

**DOI:** 10.3897/BDJ.14.e194577

**Published:** 2026-08-04

**Authors:** Rūta Starka, Solvita Rūsiņa, Liene Auniņa, Maksims Balalaikins, Jekaterīna Butkeviča, Inese Cera, Dagmāra Čakstiņa, Andris Čeirāns, Jānis Gailis, Ligita Liepiņa, Ivars Leimanis, Anna Mežaka, Rolands Moisejevs, Jānis Ozols, Digna Pilāte, Ilze Priedniece, Raitis Riters, Betija Rubene, Līga Strazdiņa, Vita Šakele, Artūrs Vancāns, Vineta Vērpēja, Viesturs Vintulis, Marks Arnolds Župerka, Ivo Vinogradovs, Ainārs Auniņš, Andris Avotiņš

**Affiliations:** 1 Department of Ecology, Faculty of Medicine and Life Sciences, University of Latvia, Riga, Latvia Department of Ecology, Faculty of Medicine and Life Sciences, University of Latvia Riga Latvia; 2 Department of Geography, Faculty of Science and Technology, University of Latvia, Riga, Latvia Department of Geography, Faculty of Science and Technology, University of Latvia Riga Latvia; 3 Institute of Biology, Faculty of Medicine and Life Sciences, University of Latvia, Riga, Latvia Institute of Biology, Faculty of Medicine and Life Sciences, University of Latvia Riga Latvia; 4 Institute of Life Sciences and Technology, Daugavpils University, Daugavpils, Latvia Institute of Life Sciences and Technology, Daugavpils University Daugavpils Latvia; 5 Department of Ecology, Institute of Life Sciences and Technology, Daugavpils University, Daugavpils, Latvia Department of Ecology, Institute of Life Sciences and Technology, Daugavpils University Daugavpils Latvia; 6 Institute of Plant Protection Research “Agrihorts”, Latvia University of Life Sciences and Technologies, Jelgava, Latvia Institute of Plant Protection Research “Agrihorts”, Latvia University of Life Sciences and Technologies Jelgava Latvia; 7 Nature Conservation Agency Republic of Latvia, Sigulda, Latvia Nature Conservation Agency Republic of Latvia Sigulda Latvia; 8 Latvian State Forest Research Institute “Silava”, Salaspils, Latvia Latvian State Forest Research Institute “Silava” Salaspils Latvia; 9 Latvian Fund for Nature, Riga, Latvia Latvian Fund for Nature Riga Latvia

**Keywords:** habitat suitability models, multispecies prioritisation, spatial planning

## Abstract

**Background:**

Achieving the goals of the EU Biodiversity Strategy for 2030 requires reliable, evidence‑based tools to guide conservation planning. Habitat suitability models can help identify priority areas by predicting suitable habitats for taxa of conservation concern. However, their usefulness depends heavily on the quality of input data. Using heterogeneous data sources, especially citizen-science occurrences, poses challenges for ensuring quality-informed use of these records. In addition, inconsistencies in the selection of environmental variables can compromise the reliability of the results, particularly when combining predictions across multiple species. Therefore, transparent species selection and rigorous, harmonised data preparation are essential for producing robust spatial assessments that can effectively support national‑level conservation decisions.

**New information:**

We present conceptual framework and methodological rationale for a structured dataset for model-based conservation planning in Latvia, from species selection and observations to species-specific ecogeographical variable selection. The framework was developed and the dataset created as a part of the state research programmes project "HiQBioDiv: High-resolution quantification of biodiversity for conservation and management". The dataset provides a basis for multi-taxon habitat suitability modelling that can be further used in site prioritisation for conservation as well as in ecological and socio-economic assessments. As a part of this dataset, we provide 162,186 expert-filtered occurrences from 464 taxa, most of which were previously unavailable to the international community.

## Introduction

The EU Biodiversity Strategy for 2030 (European Commission and Directorate-General for Environment 2020) aims to designate at least 30% of the European Union's (EU's) land area for legal protection to target nature conservation. Currently, Latvia has one of the lowest combined Natura 2000 network site area in Europe, covering approximately 12% of the country (Tucker 2023). Additionally, 90% of the EU Habitats Directive Annex I habitats and 60% of Annex II and IV species have unfavourable or unknown conservation status in Latvia (Ministry of Environmental Protection and Regional Development 2019a, Ministry of Environmental Protection and Regional Development 2019b), which is a red flag for the sustainability of the national nature capital. This highlights the urgency to assess species and habitat management and to identify territories where the status of legal protection benefits species and habitats of EU and national importance.

To achieve the targets of EU Biodiversity Strategy for 2030 (European Commission and Directorate-General for Environment 2020), it is important to develop an evidence-based, transparent decision-making process that promotes a balanced approach to biodiversity conservation. Habitat suitability modelling provides valuable insights into species ecology and potential distribution (Phillips et al. 2006, Elith et al. 2010) and is informative when planning the conservation of species and their habitats (Erickson and Smith 2023). Previous practical applications have shown that habitat suitability models of specialist species can provide information about the effectiveness of the existing protected area network and reveal new sites of high conservation value (Avotins et al. 2022). Applying the same approach to a set of organisms representing different trophic levels, ecological functions and dispersal abilities – especially when including protected, endangered and umbrella species – can enable the creation of spatially explicit predictions of national-level priority sites.

However, the usefulness of these models depends on the quality of the input data, including precise spatial placement and accurate species identification (Aubry et al. 2017). This is especially addressable if citizen-science data are used for species presences (Matutini et al. 2021). The effect of data quality may be amplified when overlaying habitat suitability predictions of multiple species. Therefore, preparation of harmonised input data, ensuring the reliability of the occurrence data and transparency of species selection, is crucial to successfully apply the model results to practical conservation.

## General description

### Purpose

This article describes the conceptual framework and methodological rationale underlying data preparation for model-based conservation planning in Latvia. It covers species selection, compilation of occurrence records and selection of environmental predictors for presence-only habitat suitability modelling.

### Additional information

The presented dataset consists of four parts:

1. a species list, where each species is evaluated using multiple criteria for being included in distribution modelling process;
2. an observation mislocation filtering guide, containing annotations of land-cover classes that are unsuitable for each species;
3. an observation file, containing expert-verified, spatially filtered observations for the selected species;
4. a selection of ecogeographical variables (Avotins et al. 2025) that can be used as environmental drivers for each species when conducting habitat suitability modelling in Latvia.

All data tables contain the following key fields: “Group” (a wider taxonomic group), “NR” (an assigned taxon number within each taxonomic group), “ScientificName” (the species scientific name) and “CODE” (a unique species acronym, usually composed of the first letters of genus and specific epithet). For each species, the combination of these fields is unique; therefore, they can be used for data filtering within each table or for cross-referencing between the tables. The other fields in each table vary and are described in the according “README” pages (field description tables).

The framework was developed and the dataset created as a part of the state research programmes project "HiQBioDiv: High-resolution quantification of biodiversity for conservation and management".

## Project description

### Title

“HiQBioDiv” - “High-resolution quantification of biodiversity for conservation and management”

### Study area description

The project is limited to the terrestrial territory of Latvia (see “Geographic coverage”).

### Design description

The project develops high‑resolution, nationwide habitat suitability models for EU‑protected taxa with unfavourable or unknown conservation status, as well as habitat specialist, indicator and umbrella species. By integrating expertise in ecology, ecological modelling and geospatial data processing, the project aims to generate spatially explicit biodiversity layers that support nature conservation planning, climate change mitigation and socio‑economic assessments. These outputs will be used to prioritise sites for species and habitat protection and to propose enhancements to the national protected area network. The project design consists of multiple work packages: data preparation and preprocessing; habitat suitability modelling and spatial conservation prioritisation; in-depth studies of EU protected species and habitats; socio-ecological analysis and evaluation; project management and result dissemination.

### Funding

This research is funded by the Ministry of Smart Administration and Regional Development, project HiQBioDiv: High-resolution quantification of biodiversity for conservation and management, No. VPP-VARAM-Daba-2024/1-0002.

## Sampling methods

### Sampling description

**The “SpeciesList” dataset** is composed of three parts – the Nomenclature table, the Species List table and the field description (README) table. These tables list all species considered for habitat suitability modelling in the project. Inclusion in the dataset is based either on a full nation-wide species list (Tracheophyta, Orthoptera, Coleoptera, Papilionoidea, Amphibia, Reptilia except *Emys orbicularis* that has a single stable population and an insufficient number of observations for modelling) (Gavrilova and Šulcs 1999, Telnov 2004, Wiemers et al. 2018, Starka et al. 2022, University of California 2025), on a list of species that are informative to nature conservation, such as protected, endangered, typical, umbrella and indicator species (Poroid fungi, Bryophyta, Diptera, Mollusca, Araneae) (Cabinet of Ministers 2000, Auniņš 2013, Meiere 2019, LIFE FOR SPECIES 2025) or on a list of species that are identifiable from photographs (Hymenoptera). As modelling species without observations is not feasible, for Papilionidea, Coleoptera and Hymenoptera, an additional criterion for inclusion in the “SpeciesList” dataset was at least one observation registered in citizen-science geoportal “Dabasdati” (Latvijas Dabas Fonds and Latvijas Ornitoloģijas biedrība 2024) since 2016. More detailed descriptions of the traits included in the Species List and Nomenclature tables are provided in the "Traits coverage” section.

Criteria for selecting a species for habitat suitability modelling were primarily based on their role in nature conservation, such as inclusion in the annexes of the EU Habitats Directive (Council of the European Union 1992), unfavourable-inadequate (U1), unfavourable-bad (U2) or unknown (XX) population or range trends or overall conservation status in Latvia (Ministry of Environmental Protection and Regional Development 2019b, Implementation and Monitoring Commission 2024), species that are listed in the Convention on the conservation of migratory species of wild animals (European Economic Community 1982) and the Convention on the conservation of European wildlife and natural habitats (Anonymous 1982), protected or micro-reserve species (Cabinet of Ministers 2000, Cabinet of Ministers 2012) or nationally Red-listed species (LIFE FOR SPECIES 2025). As rare species often have limited number of observations, species that are habitat quality, functionality or diversity indicators (Kotiranta and Niemelä 1993, Lārmanis et al. 2000, Lārmanis 2013, von Bonsdorff et al. 2014, European Environment Agency 2025) and species that are typical of EU habitat types (Auniņš 2013) were also prioritised as they can serve as umbrella species in conservation planning. The limiting factor in selecting species for modelling was the number of observations in the period 2016-2024 which was set to a minimum of 20 expert-validated observations before spatial filtering (see description of the Observations table). As for many taxonomic groups, there is no monitoring scheme in place and the main source of data was the citizen-science geoportal “Dabasdati” (Latvijas Dabas Fonds and Latvijas Ornitoloģijas biedrība 2024) or the governmental nature data management system “OZOLS” (Nature Conservation Agency 2025), an additional limitation for selecting the species for modelling was the complexity of species identification – if the species could not be confidently identified using photos, the species was not selected for modelling. Expansive, invasive, non-native, synanthropic, ecologically plastic and taxonomically challenging species were not selected for modelling, because their models were considered unlikely to provide robust or conservation-relevant habitat-suitability estimates. The classification of species as expansive, synanthropic, ecologically very plastic or difficult to identify in the field was based primarily on expert judgement. Some species that appear as selected for modelling in the Species List were not included in the Observations table, due to national restrictions aimed at protecting sensitive species from human interference (Nature Conservation Agency 2023).

**The Observations table** contains 162,186 observations from 464 taxa that were selected for modelling. The data were pulled from multiple resources – “Dabasdati” (Latvijas Dabas Fonds and Latvijas Ornitoloģijas biedrība 2024), “OZOLS” (Nature Conservation Agency 2025), the biodiversity data management platform “PlutoF” (Kõljalg et al. 2019), data collected by the Institute of Plant Protection Research “Agrihorts”, various projects (“GrassLIFE”, LIFE16NAT/LV/000262; “GrassLIFE2”, LIFE21-NAT-LV-GrassLIFE2; “LatViaNature”, LIFE19IPE/LV/000010; LIFE FOR SPECIES, LIFE19GIE/LV/000857) and personal databases. Number of observations in each species group per data source is provided in Suppl. material 1. Each observation was validated by the experts of the taxonomic group (authors of this publication) to ensure that the identification of the specimen is correct (if photo, video or sound recording were available) and there are no obvious coordinate accuracy errors. For data originating from “Dabasdati” or “OZOLS”, observations without photos were kept only if the observer is also an expert of the corresponding species group. For each observation, the source, its relative reliability category (citizen science, expert / research or mixed) and source-specific identifier were provided. The year, date and exact time of the observation were provided if available (see “Temporal coverage”). All the occurrences were in EPSG:3059 (LKS-92 / Latvia TM) coordinate system. The coordinates were rounded to species-specific resolution (100, 1000 or 5000 m grid cell centre) to avoid revealing exact locations of sensitive species or species that could be threatened by collection and spatially filtered by keeping only the last known observation per grid cell. Species (n = 10) occurrences whose disclosure is restricted under the regulation of Nature Conservation Agency (2023) were omitted from the Observations table (all Chiroptera species and *Cypripedium calceolus*). A summary of observations is provided in Table 1.

If available, the information on abundance was kept as in the original source. Otherwise, the minimum of a single observed individual was assumed. If *ad-libitum* information were available for any of the observations (such as life stage, sex, status of the observation), it is summarised in the respective fields as unique categories. The dataset also includes annotations on whether the observation has a potential coordinate location error, based on broad Corine Land Cover classes (see “Filtering Guide”).

Finally, the accumulated change of land cover since the year of observation was calculated for all observations that date from 2016 to 2024. For this calculation, two data sources were used – (1) the Global Forest Watch v1.12 tree cover loss layer, which describes the annual loss of tree canopy loss from 2001 to the current version, at a resolution of 30 m (Hansen et al. 2013) and (2) Dynamic World, which classifies land cover and land use (LULC) into nine categories (0 - water; 1 - trees; 2 - grass; 3 - flooded vegetation; 4 - crops; 5 - shrub and scrub; 6 - built; 7 - bare; 8 - snow and ice), for each ESA Copernicus Sentinel-2 image with identified cloudiness ≤ 35%, allowing for filtering and various aggregations (Brown et al. 2022). The tree cover loss layer was downloaded from the Google Earth Engine (Gorelick et al. 2017) and resampled to match the projects’ template raster grid at 10 m resolution (Avotins 2024). This layer was used to calculate the number of 10 m cells with tree cover loss indicated per every 100 m analysis raster cell and per each of the preselected landscape scales (radius of 500 m, 1250 m, 3000 m and 10000 m around the centre of the analysis cell). In the case of the Dynamic World dataset, we prepared a classification layer specific to each year, covering input data ranging from April to August and resampled the grid values to match the 10 m raster template using the nearest-neighbour method. Once the year-specific layer was obtained, we calculated the number of 10 m pixels with a land-cover class change per 100 m analysis grid cell and for each of the preselected landscape scales (radius of 500 m, 1250 m, 3000 m and 10000 m around the centre of the analysis cell). Once the yearly values had been calculated, we accumulated dataset-specific values at the analysis grid cell resolution and species-specific home range scale over the time period from the year of observation until 2024 and added to the observations table.

**The Filtering Guide** dataset contains all taxa selected for modelling and shows the approach to filtering the observations for possible location errors, based on broad landscape classes, as well as annotations for the necessity of land-use land-cover (LULC) change analysis. Corine Land Cover classes (Copernicus Land Monitoring Service 2020) are combined to describe broader groups: built up areas (CLC codes 111, 112, 121, 122, 123, 124, 131, 132, 133, 142), open landscape (CLC codes 211, 222, 231, 242, 243, 321, 322, 331), areas covered by trees (CLC codes 141, 311, 312, 313, 324, 333), wetlands (CLC codes 411, 412) and water (CLC codes 511, 512, 523). As some species occur in costal dunes, potential mislocations in the sea were denoted using Latvia’s Exclusive Economic Zone waters and the coastline of the Baltic Sea files obtained from the HELCOM map and data service (HELCOM Secretariat 2019) that offers higher resolution than CLC class 523. If an observation were in a land-cover group that is indicated with “-1” for the species in the Filtering Guide, this observation was considered a potential location error in the Observations table (fields “Mislocation_CLC” and “Mislocation_Sea”). Finally, annotations whether LULC change has already been performed for the species observations (field “StatusLULCchangeAnalysis”) and for which landscape classes should the LULC changes be calculated (field “EnvironmentLULCchangeAnalysis”) (see “Observations”) were introduced.

For all taxa selected for modelling in the project, a selection of ecogeographical variables (EGVs) for habitat suitability modelling was suggested in **the EGV table**. The selection for each species was based on the species biology and ecology, following a unified approach that is described below. We used a publicly available dataset (Avotins et al. 2025) containing 538 EGVs at a resolution of 100 m, describing:

- climate (69 variables);
- hydrology (19 variables);
- terrain (13 variables);
- soil (26 variables);
- Earth observation products (21 variables);
- landscape diversity and composition (82 variables);
- agriculture (90 variables);
- forestry (133 variables);
- distance to landscape features (9 variables);
- other land-cover classes (76 variables).

Some of the variables were calculated only at a resolution of 100 m (climate, Earth observation products, forestry quantities, distance to landscape features and terrain variables), while others were calculated at multiple spatial scales created as buffers around the centre of every 100 m raster grid cell. The radii used were 500 m, 1250 m, 3000 m and 10000 m.

All the variables were considered for each species. Variables that were calculated only at cell scale were picked so that only the most meaningful from sets of variables describing the same environmental conditions in different terms was selected, prioritising variables that have a clear expected biological response as suggested by the species experts (hypothesis-based selection). Climate variables were assigned according to each species biology and distribution, selecting more climate variables for species at the border of their distribution range, as climate often is the main large scale driver of distributions.

For EGVs that were calculated at multiple scales (100 m, 500 m, 1250 m, 3000 m and 10000 m), three scales were consequently used for each species – cell (100 m resolution), home range (see description of the Species List table; indicated by the authors to best match the scales of EGVs and assumed to be 500 m for sedentary organisms and species with very small home ranges) and landscape scale (either 1250, 3000 or 10000 m, whichever is one step higher than the home range, to characterise the landscape context around the species observations). To reduce the number of variables that would be excluded from the species habitat suitability modelling due to multicollinearity and spatial autocorrelation, we avoided assigning variables that carried the same information at multiple scales. For the same reasons and to enable the model results to capture small-scale heterogeneity, we assigned more specific EGVs at smaller scales, increasing the level of generalisation with the spatial scale. For example, for a forest-dwelling species, we used forest age, timber volume and similar variables at the 100 m resolution, more general variables such as fractional cover of different tree age classes at the home range scale and even more general fractional cover of forests supplemented by, for example, fractional cover of different forest growing condition classes at the landscape scale.

We respected the gradient of habitat suitability when selecting the variables to avoid stereotyping species ecology. For example, if a species is known as an old-growth forest specialist, a single fractional cover of old forests was not assumed - rather fractional cover of all age classes were selected to learn the associations via habitat suitability models. For environment that is unsuitable for the species, all scales from the “General” class were used (see EGV repository description in Avotins et al. (2025)).

## Geographic coverage

### Description

**Figure 1.**
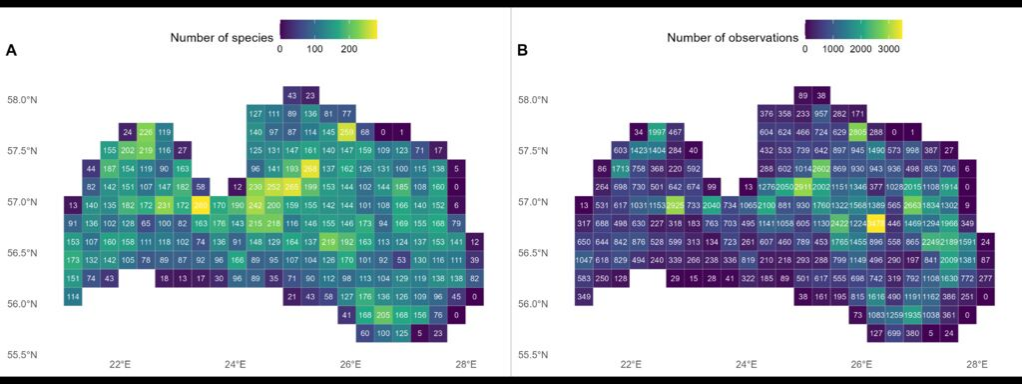
Spatial coverage of the dataset on a 20 km grid. A - number of species with observations in each grid cell; B - total number of observations in each grid cell.

The occurrence data cover the terrain territory of Latvia (Fig. 1) – a northern European country with the area of 64573 km^2^ (Nikodemus et al. 2018). Latvia is located in the lowest region of the Eastern European Plain, with the average elevation of 89.5 metres and the minimum and maximum elevation ranging from 0 to 311.9 metres above sea level (Nikodemus et al. 2018). The climate is influenced by the proximity to the Baltic Sea and Gulf of Riga which reduces temperature fluctuations, making coastal winters milder and summers cooler, while continentality increases eastwards (Nikodemus et al. 2018). The average air temperature varies from -1.6°C to -5.8°C in February and from +16.8°C to +18.8°C in July and the average annual amount of precipitation is 709 mm (Nikodemus et al. 2018).

### Coordinates

55.674971 and 58.085137 Latitude; 20.970154 and 28.241043 Longitude.

## Taxonomic coverage

### Description

The dataset covers multiple major taxonomic groups – vertebrates (Amphibia, Reptilia, Chiroptera), invertebrates (Araneae, Mollusca, Insecta: Coleoptera, Diptera, Hymenoptera, Orthoptera, Papilionoidea), forest, wetland and grassland vascular plants (Tracheophyta) as well as mosses and liverworts (Bryophyta), lichens and poroid fungi. The dominant taxonomic level in the dataset is species. In two cases, *Pelophylax* sp. (Amphibia) and *Anguis* sp. (Reptilia), the distinction level is genus, as species identification relies on molecular methods. In the Tracheophyta group, hybrids and subspecies are also represented. The number of nomenclature units and species per group is summarised in Table 1. The use of species scientific names is based on GBIF Backbone Taxonomy (GBIF Secretariat 2023) for Diptera, Hymenoptera, Araneae, Mollusca, Amphibia, Reptilia and Chiroptera; “Dabasdati” (Latvijas Dabas Fonds and Latvijas Ornitoloģijas biedrība 2024) and GBIF Backbone Taxonomy (GBIF Secretariat 2023) for Coleoptera; the Orthoptera Species File taxonomic database (Cigliano et al. 2025) for Orthoptera; Wiemers et al. (2018) for Papilionoidea; Randlane et al. (2025) for lichens; Meiere (2019) for poroid fungi; Hodgetts et al. (2020) for Bryophyta; and Gavrilova and Šulcs (1999) and the national Red List (LIFE FOR SPECIES 2025) for Tracheophyta. Additionally, the common names of the species, if existing, are provided in the Nomenclature table. The sources of common names for each taxonomic group are provided in the associated field description sheet (README).

## Traits coverage

**The Nomenclature table** contain species names as they appear in relevant legislation documents, such as the EU Habitats Directive (Council of the European Union 1992), Bonn Convention (European Economic Community 1982), Bern Convention (Anonymous 1982) and the national report to the European Commission on these documents (Ministry of Environmental Protection and Regional Development 2019b), the list of specially protected species in Latvia (Cabinet of Ministers 2000) and the list of specially protected species for which micro-reserves can be established (Cabinet of Ministers 2012, Annex 1). Additionally, here species names were compiled as they were used in the Annex 12 of the project competition regulation (Implementation and Monitoring Commission 2024), the national Red List (LIFE FOR SPECIES 2025), lists of natural habitat indicator species (Lārmanis et al. 2000, Lārmanis 2013), EU habitat type typical and umbrella species (Auniņš 2013) and other group-specific lists and information sources (Kotiranta and Niemelä 1993, von Bonsdorff et al. 2014, Mucina et al. 2016, Meiere 2019, European Environment Agency 2025, Euro+Med PlantBase 2025). Overall, 4215 nomenclature units from 4176 taxa were included.

Traits that characterise each species importance in nature conservation are covered by the **Species List table**. It contains annotations on each taxa inclusion in the abovementioned legislation documents and species lists (1 – yes, 0 – no), as well as character fields, such as the national Red List IUCN categories (LIFE FOR SPECIES 2025), conservation status (Ministry of Environmental Protection and Regional Development 2019b), as well as expert-based considerations that limit the opportunity to model the species habitat suitability. The table also contains counts of 100 m and 1 km grid cells with at least one observation in nature data management system “OZOLS” (Nature Conservation Agency 2025) and “Dabasdati” (Latvijas Dabas Fonds and Latvijas Ornitoloģijas biedrība 2024) databases since 2016; however, as these are online databases that are updated regularly, these numbers are subject to changes (data from "OZOLS" was acquired on 25/02/2025 and on 20/12/2024 from "Dabasdati"). For each species, a home range radius approximation is suggested by the authors (best matching to one of the existing scales of EGVs), which is useful when choosing ecogeographical variables and adapting distribution modelling parameters. Finally, the table contains annotation on whether the species is selected for modelling in the project and the resolution at which the species observations and distribution projections can be published, complying with the restrictions set by the regulation of Nature Conservation Agency (2023).

## Temporal coverage

**Living time period:** 2000 - 2025.

### Notes

The Observations table includes observations from 2000 to 2025, although most records date from 2016 to 2024. Observations from 2000 - 2015 and from 2025 were included in the modelling for rare and protected species with very limited number of records; however, these observations do not have calculations of environmental changes since the year of the observation (see description of the Observations table). The observation year is available for all records. Most records (99.5%) come with an observation date and a small fraction (13.2%) also have observation time. System-defined time signatures are removed.

## Usage licence

### Usage licence

Other

### IP rights notes

CC BY-NC 4.0

## Data resources

### Data package title

Species dataset for model-based conservation planning in Latvia: occurrences, importance for nature conservation planning and ecogeographical variable selection

### Resource link

https://zenodo.org/records/ 19113200

### Alternative identifiers

https://doi.org/10.5281/zenodo. 19113200

### Number of data sets

5

### Data set 1.

#### Data set name

SpeciesList

#### Data format

xlsx

#### Data format version

csv

#### Description

Annotations and decision‑making criteria used when selecting species for distribution modelling.

**Data set 1.** 

Column label	Column description
Group	Taxonomic group.
NR	Assigned taxon number within group.
ScientificName	Scientific name.
CODE	Species specific code used in this project.
RegulationAnnex12	Indication of whether the species is listed in Implementation and Monitoring Commission (2024) Regulations for the Open Call for Proposals of the National Research Programme “Development of Research Identified in the Biodiversity Priority Actions Programme”. URL: https://www.lzp.gov.lv/lv/media/7650/download?attachment: '1' - yes, '0' - no.
HabitatsDirective	Indication of whether the species is listed in Council of the European Union (1992) Council Directive 92/43/EEC of 21 May 1992 on the conservation of natural habitats and of wild fauna and flora. Official Journal of the European Communities 206: 7‑50. URL: http://data.europa.eu/eli/dir/1992/43/oj: '1' - yes, '0' - no.
BonnConvention	Indication of whether the species is listed in European Economic Community (1982) Convention on the conservation of migratory species of wild animals (Bonn Convention). Official Journal of the European Communities 210: 11‑22. URL: http://data.europa.eu/eli/convention/1982/461/oj: '1' - yes, '0' - no.
BernConvention	Indication of whether the species is listed in Anonymous (1982) Convention on the conservation of European wildlife and natural habitats (Bern Convention). Official Journal of the European Communities 38: 3‑32. URL: http://data.europa.eu/eli/convention/1982/72/oj: '1' - yes, '0' - no.
ProtectedSpeciesLV	Indication of whether the species is listed in Cabinet of Ministers (2000) Cabinet of Ministers Regulation No. 396 "Noteikumi par īpaši aizsargājamo sugu un ierobežoti izmantojamo īpaši aizsargājamo sugu sarakstu" (Regulation on the list of specially protected and restricted use specially protected species). Latvijas Vēstnesis, 413/417, 17.11.2000. (In Latvian). URL: https://likumi.lv/ta/id/12821: '1' - yes, '0' - no.
MicroreserveSpeciesLV	Indication of whether the species is listed in Cabinet of Ministers (2012) Cabinet of Ministers Regulation No. 940 “Regulations Regarding the Establishment and Management of Micro-reserves, Their Conservation, as well as Determination of Micro-reserves and Their Buffer Zones” Annex 1 “Species of Specially Protected Mammals, Amphibians, Reptiles, Invertebrates, Vascular Plants, Moss, Algae, Lichen and Fungus for which Micro-reserves shall be Established”. Latvijas Vēstnesis, 203, 28.12.2012. URL: https://likumi.lv/ta/en/en/id/253746-regulationsregarding-the-establishment-and-management-of-micro-reserves-their-conservation-aswell-as-determination-of-micro-reserves-and-their-buffer-zones: '1' - yes, '0' - no.
EndangeredSpecies	Red List assesment from project LIFE FOR SPECIES (2025) Threatened species in Latvia: improved knowledge, capacity, data and awareness. Red List assessment, version 09/10/2025. LIFE project LIFE FOR SPECIES (Project No. LIFE19GIE/LV/000857). URL: https://sarkanagramata.lu.lv/en/news/the-electronic-edition-of-the-new-latvian-red-data-book/; Red List category or empty if species is not evaluated.
PotentiallyProtectedSpecies	Assessment for listing as protected species from project LIFE FOR SPECIES (2025) Threatened species in Latvia: improved knowledge, capacity, data and awareness. Red List assessment, version 09/10/2025. LIFE project LIFE FOR SPECIES (Project No. LIFE19GIE/LV/000857). URL: https://sarkanagramata.lu.lv/en/news/the-electronic-edition-of-the-new-latvian-red-data-book/; '1' - yes, '0' - no.
ConservationStatus	As reported for the period 2013-2018 in the report on the Ministry of Environmental Protection and Regional Development (2019b) Article 17 Report under the Habitats Directive (92/43/EEC): Main results of the surveillance of Annex II, IV & V species pursuant to Article 11 for the period 2013–2018. Latvia. Submitted to the European Commission, 2019. URL: https://cdr.eionet.europa.eu/Converters/run_conversion?file=lv/eu/art17/envxwalvg/LV_species_reports-20190829-115440.xml&conv=593&source=remote#1951; empty otherwise.
PopulationSTTperiod	Period for the short-term population trend as in Ministry of Environmental Protection and Regional Development (2019b) Article 17 Report under the Habitats Directive (92/43/EEC): Main results of the surveillance of Annex II, IV & V species pursuant to Article 11 for the period 2013–2018. Latvia. Submitted to the European Commission, 2019. URL: https://cdr.eionet.europa.eu/Converters/run_conversion?file=lv/eu/art17/envxwalvg/LV_species_reports-20190829-115440.xml&conv=593&source=remote#1951; empty if species is not listed in EU Nature Directives.
PopulationSTTdirection	Direction of the short-term population trend as in Ministry of Environmental Protection and Regional Development (2019b) Article 17 Report under the Habitats Directive (92/43/EEC): Main results of the surveillance of Annex II, IV & V species pursuant to Article 11 for the period 2013–2018. Latvia. Submitted to the European Commission, 2019. URL: https://cdr.eionet.europa.eu/Converters/run_conversion?file=lv/eu/art17/envxwalvg/LV_species_reports-20190829-115440.xml&conv=593&source=remote#1951; empty if species is not listed in EU Nature Directives.
PopulationLTTperiod	Period of the long-term population trend as in Ministry of Environmental Protection and Regional Development (2019b) Article 17 Report under the Habitats Directive (92/43/EEC): Main results of the surveillance of Annex II, IV & V species pursuant to Article 11 for the period 2013–2018. Latvia. Submitted to the European Commission, 2019. URL: https://cdr.eionet.europa.eu/Converters/run_conversion?file=lv/eu/art17/envxwalvg/LV_species_reports-20190829-115440.xml&conv=593&source=remote#1951; empty if species is not listed in EU Nature Directives or if long-term trend period is not reported.
PopulationLTTdirection	Direction of the long-term population trend as in Ministry of Environmental Protection and Regional Development (2019b) Article 17 Report under the Habitats Directive (92/43/EEC): Main results of the surveillance of Annex II, IV & V species pursuant to Article 11 for the period 2013–2018. Latvia. Submitted to the European Commission, 2019. URL: https://cdr.eionet.europa.eu/Converters/run_conversion?file=lv/eu/art17/envxwalvg/LV_species_reports-20190829-115440.xml&conv=593&source=remote#1951; empty if species is not listed in EU Nature Directives or if long-term trend direction is not reported.
RangeSTTperiod	Period of the short-term distribution trend as in Ministry of Environmental Protection and Regional Development (2019b) Article 17 Report under the Habitats Directive (92/43/EEC): Main results of the surveillance of Annex II, IV & V species pursuant to Article 11 for the period 2013–2018. Latvia. Submitted to the European Commission, 2019. URL: https://cdr.eionet.europa.eu/Converters/run_conversion?file=lv/eu/art17/envxwalvg/LV_species_reports-20190829-115440.xml&conv=593&source=remote#1951; empty if species is not listed in EU Nature Directives.
RangeSTTdirection	Direction of the short-term distribution trend as in Ministry of Environmental Protection and Regional Development (2019b) Article 17 Report under the Habitats Directive (92/43/EEC): Main results of the surveillance of Annex II, IV & V species pursuant to Article 11 for the period 2013–2018. Latvia. Submitted to the European Commission, 2019. URL: https://cdr.eionet.europa.eu/Converters/run_conversion?file=lv/eu/art17/envxwalvg/LV_species_reports-20190829-115440.xml&conv=593&source=remote#1951; empty if species is not listed in EU Nature Directives.
RangeLTTperiod	Period of the long-term distribution trend as in Ministry of Environmental Protection and Regional Development (2019b) Article 17 Report under the Habitats Directive (92/43/EEC): Main results of the surveillance of Annex II, IV & V species pursuant to Article 11 for the period 2013–2018. Latvia. Submitted to the European Commission, 2019. URL: https://cdr.eionet.europa.eu/Converters/run_conversion?file=lv/eu/art17/envxwalvg/LV_species_reports-20190829-115440.xml&conv=593&source=remote#1951; empty if species is not listed in EU Nature Directives or if long-term trend period is not reported.
RangeLTTdirection	Direction of the long-term distribution trend as in Ministry of Environmental Protection and Regional Development (2019b) Article 17 Report under the Habitats Directive (92/43/EEC): Main results of the surveillance of Annex II, IV & V species pursuant to Article 11 for the period 2013–2018. Latvia. Submitted to the European Commission, 2019. URL: https://cdr.eionet.europa.eu/Converters/run_conversion?file=lv/eu/art17/envxwalvg/LV_species_reports-20190829-115440.xml&conv=593&source=remote#1951; empty if species is not listed in EU Nature Directives or if long-term trend direction is not reported.
NFHspecialistSpecies	Indication of whether the species is listed in Lārmanis V, Priedītis N, Rudzīte M (2000) Mežaudžu atslēgas biotopu rokasgrāmata. (Handbook of natural forest habitats). Valsts meža dienests, Rīga, 127 pp. (In Latvian) as natural forest habitat indicator species: '1' - yes, '0' - no.
HabitatIndicatorSpecies	Indication of whether the species is listed in Auniņš A (Ed.) (2013) European Union Protected Habitats in Latvia. Interpretation Manual. 2nd revised edition. Latvian Fund of Nature, Ministry of Environmental Protection and Regional Development, Rīga, 320 pp. URL: https://www.daba.gov.lv/lv/media/5945/download?attachment as habitat indicator species: '1' - yes, '0' - no.
HNVgrasslandSpecies	Indication of whether the species is listed in Lārmanis V (Ed.) (2013) Bioloģiski vērtīgo zālāju kartēšanas metodika. (Methodology for mapping high nature value grasslands). Dabas aizsardzības pārvalde, Sigulda, 61 pp. (n Latvian). URL: https://ppdb.mk.gov.lv/wp-content/uploads/2023/04/Kartesanas-metodika.pdf as high nature value grassland indicator species: '1' - yes, '0' - no.
CharacteristicUmbrellaSpecies	Indication of whether the species is listed in Auniņš A (Ed.) (2013) European Union Protected Habitats in Latvia. Interpretation Manual. 2nd revised edition. Latvian Fund of Nature, Ministry of Environmental Protection and Regional Development, Rīga, 320 pp. URL: https://www.daba.gov.lv/lv/media/5945/download?attachment chapters depicting indicator and umbrella species: '1' - yes, '0' - no.
GrasslandButterflyIndex	Indication of whether the species is listed in European Environment Agency (2025) Grassland butterfly index in Europe. https://www.eea.europa.eu/en/analysis/indicators/grassland-butterfly-index-in-europe-1?activeAccordion=546a7c35-9188-4d23-94ee-005d97c26f2b. Accessed on: 01-02-2025.; '1' - yes, '0' - no; empty if not applicable.
Kotiranta_Niemelä_1993	Indication of wether species is listed as an indicator in: 1. Kotiranta H, Niemelä T (1993) Uhanalaiset käävät Suomessa. (Threatened polypores in Finland). Vesi- ja ympäristöhallinnon julkaisuja - sarja B; 17. Finnish Water and Environment Agency, Helsinki, 116 pp. (In Finnish). URL: https://helda.helsinki.fi/items/c35f215a-3363-4103-b951-3a2ca67c696e (ISBN 95147-7679-1); 2. von Bonsdorff T, Kytövuori I, Vauras J, Huhtinen S, Halme P, Rämä T, Kosonen L, Jakobsson S (2014) Sienet ja metsien luontoarvot (Fungi as indicators of conservation value of forests). Norrlinia 27: 1‑272. (In Finnish): '1' - listed as indicator in the first source, '2'- listed as a higher importance indicator in the second source, '*' - indicator of virgin forests; empty if not listed in either of the sources.
Grid1km_Dabasdati	Number of 1 km grid cells with at least one registered observation in Latvijas Dabas Fonds, Latvijas Ornitoloģijas biedrība (2024) Dabasdati.lv. https://dabasdati.lv/lv since 01-01-2016; empty if expert compiled own observation database.
Grid100m_Dabasdati	Number of 100 m grid cells with at least one registered observation in Latvijas Dabas Fonds, Latvijas Ornitoloģijas biedrība (2024) Dabasdati.lv. https://dabasdati.lv/lv since 01-01-2016; empty if expert compiled own observation database.
Grid1km_OZOLS	Number of 1 km grid cells with at least one registered observation in Nature Conservation Agency (2025) Nature Data Management System OZOLS. https://www.daba.gov.lv/en/nature-data-management-system-ozols since 01-01-2016; empty if expert compiled own observation database.
Grid100m_OZOLS	Number of 100 m grid cells with at least one registered observation in Nature Conservation Agency (2025) Nature Data Management System OZOLS. https://www.daba.gov.lv/en/nature-data-management-system-ozols since 01-01-2016; empty if expert compiled own observation database.
Status	Status of the observation; empty if unknown. For Chiroptera population status in Latvia: 'B' - breeding, 'W' - wintering, 'M' - migrating and combinations of any of previously mentioned; empty otherwise.
Limitations	Assumptions limiting habitat suitability modelling for the species.
Priority	Experts decision (arbitrary) on the priority of species for distribution modelling: '-999' - not to model, otherwise lower number indicates higher priority.
HomeRange	Home range radius approximation, best matching to any of groups: '500' - up to 500 m, '1250' - up to 1250 m, '3000' - around 3000 m or more.
LandscapeScale	The radius used to characterise the landscape context around the species observations, either '1250' - up to 1250 m, '3000' - around 3000 m; '10000' - around 10000 m or more.
SelectedForModelling	Decision on habitat suitability modelling: '1' - to model, '0' - not to model.
ObservationResolution	Resolution used for publishing species observations, with '100' indicating 100 m cell centre, '1000' indicating 1000 m cell centre, '5000' indicating 5000 m cell centre, '-999' indicating that publishing species observations is prohibited according to local legislation (Nature Conservation Agency (2023) Rīkojums Nr.1.1/249/2023 “Par ierobežotas pieejamības informācijas statusa noteikšanu informācijai par īpaši aizsargājamo sugām un to dzīvotnēm”. (Regulation No. 1/249/2023 “On determining the status of restricted access information concerning specially protected species and their habitats”). Dabas aizsardzības pārvalde, 27.12.2023. (In Latvian)).
ProjectionResolution	Resolution to use when publishing species distribution habitat suitability models prediction maps, with '0' indicating highest possible and '-999' indicating that publishing species habitat suitability models is prohibited according to local legislation (Nature Conservation Agency (2023) Rīkojums Nr.1.1/249/2023 “Par ierobežotas pieejamības informācijas statusa noteikšanu informācijai par īpaši aizsargājamo sugām un to dzīvotnēm”. (Regulation No. 1/249/2023 “On determining the status of restricted access information concerning specially protected species and their habitats”). Dabas aizsardzības pārvalde, 27.12.2023. (In Latvian)).

### Data set 2.

#### Data set name

Nomenclature

#### Data format

xlsx (as the second sheet the SpeciesList.xlsx file)

#### Data format version

csv

#### Description

Species names as they appear in relevant legislation documents, species lists and other group-specific information sources.

**Data set 2.** 

Column label	Column description
Group	Taxonomic group.
NR	Assigned taxon number within group.
CommonName	Common name (in Latvian) as recognised by the expert except: for Bryophyta as used in Bambe B, Gerra-Inohosa L, Kluša J, Kukāre I, Ķeire L, Leimanis I, Liepiņa L, Long D, Mežaka A, Oļehnoviča E, Opmanis A, Pošiva-Bunkovska A, Strazdiņa L, Suško U, Fonteina-Kazeka M, Wolski G J, Zvejniece E (2023) Checklist of Latvian bryophytes. Mežaka A, Liepiņa L (eds.). Daugavpils University Press Saule, Daugavpils, 48 pp.; for Hymenoptera as used in Latvijas Dabas Fonds, Latvijas Ornitoloģijas biedrība (2025) Dabasdati.lv. https://dabasdati.lv/lv (version 03.02.2025); for Lichens as used in Dr.hab. Alfons Piterāns unpublished materials and project LIFE FOR SPECIES (2025) Threatened species in Latvia: improved knowledge, capacity, data and awareness. Red List assessment, version 09/10/2025. LIFE project LIFE FOR SPECIES (Project No. LIFE19GIE/LV/000857). URL: https://sarkanagramata.lu.lv/en/news/the-electronic-edition-of-the-new-latvian-red-data-book/; for PoroidFungi as used in Meiere D (2019) Īpaši aizsargājamās un reti sastopamās sēņu sugas Latvijā (Specially protected and rare fungi species in Latvia). LVAF project “Dabas aizsardzības pārvaldes kapacitātes stiprināšana, nodrošinot jaunu sugu aizsardzības jomas ekspertu apmācību un paaugstinot profesionālo kompetenci DAP speciālistiem” (Project No. 1-08/ 171 / 2017). (In Latvian). URL: https://www.daba.gov.lv/lv/media/8725/download?attachment; for Tracheophyta as used in Priedītis N (2025), Augi (In: Kļaviņš A, Gandrs SIA, Sugu Enciklopēdija "Latvijas Daba" (version 03.02.2025), URL: https://www.latvijasdaba.lv/augi/.
ScientificName	Scientific name as recognised by the expert except: for Bryophyta as used in Hodgetts NG, Söderström L, Blockeel TL, Caspari S, Ignatov MS, Konstantinova NA, Lockhart N, Papp B, Schröck C, Sim-Sim M, Bell D, Bell NE, Blom HH, Bruggeman-Nannenga MA, Brugués M, Enroth J, Flatberg KI, Garilleti R, Hedenäs L, Holyoak DT, Hugonnot V, Kariyawasam I, Köckinger H, Kučera J, Lara F, Porley RD (2020) An annotated checklist of bryophytes of Europe, Macaronesia and Cyprus. Journal of Bryology 42 (1): 1‑116. https://doi.org/10.1080/03736687.2019.1694329; for Diptera and Hymenoptera as used in GBIF Secretariat (2023) GBIF Backbone Taxonomy. Checklist dataset. https://doi.org/10.15468/39omei. Accessed on: 24-03-2026; for Lichens as used in: 1. Randlane T, Degtjarenko P, Jüriado I, Marmor-Ohtla L, Oja E, Saag A (2025) Distribution and Frequency of Estonian Lichens – Revisited After 25 Years. Acta Botanica Hungarica 67: 161‑172. https://doi.org/10.1556/034.67.2025.1-3.11; 2. IndexFungorum (2025). Published on the Internet https://www.indexfungorum.org, The Royal Botanic Gardens, Kew. Accessed on: 01-09-2025; for Orthoptera as used in Cigliano MM, Braun H, Eades DC, Otte D (2025) Orthoptera Species File. http://orthoptera.speciesfile.org/. Accessed on: 23-10-2025; for Papilionoidea as used in Wiemers M, Balletto E, Dincă V, Fric ZF, Lamas G, Lukhtanov V, Munguira M, van Swaay C , Vila R, Vliegenthart A, Wahlberg N, Verovnik R (2018) An updated checklist of the European Butterflies (Lepidoptera, Papilionoidea). ZooKeys 811: 9‑45. https://doi.org/10.3897/zookeys.811.28712; for PoroidFungi as used in Meiere D (2019) Īpaši aizsargājamās un reti sastopamās sēņu sugas Latvijā (Specially protected and rare fungi species in Latvia). LVAF project “Dabas aizsardzības pārvaldes kapacitātes stiprināšana, nodrošinot jaunu sugu aizsardzības jomas ekspertu apmācību un paaugstinot profesionālo kompetenci DAP speciālistiem” (Project No. 1-08/ 171 / 2017). (In Latvian). URL: https://www.daba.gov.lv/lv/media/8725/download?attachment; for Tracheophyta as used in Gavrilova Ģ, Šulcs V (1999) Latvijas vaskulāro augu flora. Taksonu saraksts. (Flora of vascular plants of Latvia. List of taxa). Latvijas Akadēmiskā bibliotēka, Rīga, 136 pp. (In Latvian).
CODE	Species specific code used in this project.
RegulationAnnex12	Species names as used in Implementation and Monitoring Commission (2024) Regulations for the Open Call for Proposals of the National Research Programme “Development of Research Identified in the Biodiversity Priority Actions Programme”. URL: https://www.lzp.gov.lv/lv/media/7650/download?attachment; empty otherwise.
HabitatsDirective	Species names as used in Council of the European Union (1992) Council Directive 92/43/EEC of 21 May 1992 on the conservation of natural habitats and of wild fauna and flora. Official Journal of the European Communities 206: 7‑50. URL: http://data.europa.eu/eli/dir/1992/43/oj; empty otherwise.
BonnConvention	Species names as used in European Economic Community (1982) Convention on the conservation of migratory species of wild animals (Bonn Convention). Official Journal of the European Communities 210: 11‑22. URL: http://data.europa.eu/eli/convention/1982/461/oj; empty otherwise.
BernConvention	Species names as used in Anonymous (1982) Convention on the conservation of European wildlife and natural habitats (Bern Convention). Official Journal of the European Communities 38: 3‑32. URL:http://data.europa.eu/eli/convention/1982/72/oj; empty otherwise.
ProtectedSpeciesLV	Species names as used in Cabinet of Ministers (2000) Cabinet of Ministers Regulation No. 396 "Noteikumi par īpaši aizsargājamo sugu un ierobežoti izmantojamo īpaši aizsargājamo sugu sarakstu" (Regulation on the list of specially protected and restricted use specially protected species). Latvijas Vēstnesis, 413/417, 17.11.2000. (In Latvian). URL: https://likumi.lv/ta/id/12821; empty otherwise.
MicroreserveSpeciesLV	Species names as used in Cabinet of Ministers (2012) Cabinet of Ministers Regulation No. 940 “Regulations Regarding the Establishment and Management of Micro-reserves, Their Conservation, as well as Determination of Micro-reserves and Their Buffer Zones” Annex 1 “Species of Specially Protected Mammals, Amphibians, Reptiles, Invertebrates, Vascular Plants, Moss, Algae, Lichen and Fungus for which Micro-reserves shall be Established”. Latvijas Vēstnesis, 203, 28.12.2012. URL: https://likumi.lv/ta/en/en/id/253746-regulationsregarding-the-establishment-and-management-of-micro-reserves-their-conservation-aswell-as-determination-of-micro-reserves-and-their-buffer-zones; empty otherwise.
LifeForSpecies	Species names as used in LIFE FOR SPECIES (2025) Threatened species in Latvia: improved knowledge, capacity, data and awareness. Red List assessment, version 09/10/2025. LIFE project LIFE FOR SPECIES (Project No. LIFE19GIE/LV/000857). URL: https://sarkanagramata.lu.lv/en/news/the-electronic-edition-of-the-new-latvian-red-data-book/; empty otherwise.
NFHspecialistSpecies	Species names as used in Lārmanis V, Priedītis N, Rudzīte M (2000) Mežaudžu atslēgas biotopu rokasgrāmata. (Handbook of natural forest habitats). Valsts meža dienests, Rīga, 127 pp. (In Latvian); empty otherwise.
HabitatIndicatorSpecies	Species names as used in Auniņš A (Ed.) (2013) European Union Protected Habitats in Latvia. Interpretation Manual. 2nd revised edition. Latvian Fund of Nature, Ministry of Environmental Protection and Regional Development, Rīga, 320 pp. URL: https://www.daba.gov.lv/lv/media/5945/download?attachment; empty otherwise.
HNVgrasslandSpecies	Species names as used in Lārmanis V (Ed.) (2013) Bioloģiski vērtīgo zālāju kartēšanas metodika. (Methodology for mapping high nature value grasslands). Dabas aizsardzības pārvalde, Sigulda, 61 pp. (In Latvian). URL: https://ppdb.mk.gov.lv/wp-content/uploads/2023/04/Kartesanas-metodika.pdf; empty otherwise.
CharacteristicUmbrellaSpecies	Species names as used in Auniņš A (Ed.) (2013) European Union Protected Habitats in Latvia. Interpretation Manual. 2nd revised edition. Latvian Fund of Nature, Ministry of Environmental Protection and Regional Development, Rīga, 320 pp. URL: https://www.daba.gov.lv/lv/media/5945/download?attachment chapters depicting characteristic and umbrella species; empty otherwise.
NatureDirectivesReport_2019	Species names as used in Ministry of Environmental Protection and Regional Development (2019b) Article 17 Report under the Habitats Directive (92/43/EEC): Main results of the surveillance of Annex II, IV & V species pursuant to Article 11 for the period 2013–2018. Latvia. Submitted to the European Commission, 2019. URL: https://cdr.eionet.europa.eu/Converters/run_conversion?file=lv/eu/art17/envxwalvg/LV_species_reports-20190829-115440.xml&conv=593&source=remote#1951; empty otherwise.
GrasslandButterflyIndex	Species names as used in European Environment Agency (2025) Grassland butterfly index in Europe. https://www.eea.europa.eu/en/analysis/indicators/grassland-butterfly-index-in-europe-1?activeAccordion=546a7c35-9188-4d23-94ee-005d97c26f2b. Accessed on: 01-02-2025; empty otherwise.
Meiere_2019	Species name as used in Meiere D (2019) Īpaši aizsargājamās un reti sastopamās sēņu sugas Latvijā (Specially protected and rare fungi species in Latvia). LVAF project “Dabas aizsardzības pārvaldes kapacitātes stiprināšana, nodrošinot jaunu sugu aizsardzības jomas ekspertu apmācību un paaugstinot profesionālo kompetenci DAP speciālistiem” (Project No. 1-08/ 171 / 2017). (In Latvian). URL: https://www.daba.gov.lv/lv/media/8725/download?attachment; empty otherwise.
Kotiranta_Niemelä_1993	Species name as used in: 1. Kotiranta H, Niemelä T (1993) Uhanalaiset käävät Suomessa. (Threatened polypores in Finland). Vesi- ja ympäristöhallinnon julkaisuja - sarja B; 17. Finnish Water and Environment Agency, Helsinki, 116 pp. (In Finnish). URL: https://helda.helsinki.fi/items/c35f215a-3363-4103-b951-3a2ca67c696e (ISBN 95147-7679-1); 2. von Bonsdorff T, Kytövuori I, Vauras J, Huhtinen S, Halme P, Rämä T, Kosonen L, Jakobsson S (2014) Sienet ja metsien luontoarvot (Fungi as indicators of conservation value of forests). Norrlinia 27: 1‑272. (In Finnish); empty otherwise.
Euro_Med_PlantBase	Species names as used in Euro+Med PlantBase (2025) Euro+Med PlantBase - the information resource for Euro-Mediterranean plant diversity. https://europlusmed.org/. Accessed on: 26-09-2025; empty otherwise.
Mucina_et_al_2016	Species names as used in Mucina L, Bültmann H, Dierßen K, Theurillat J, Raus T, Čarni A, Šumberová K, Willner W, Dengler J, García RG, Chytrý M, Hájek M, Di Pietro R, Iakushenko D, Pallas J, Daniëls FA, Bergmeier E, Santos Guerra A, Ermakov N, Valachovič M, Schaminée JJ, Lysenko T, Didukh Y, Pignatti S, Rodwell J, Capelo J, Weber H, Solomeshch A, Dimopoulos P, Aguiar C, Hennekens S, Tichý L (2016) Vegetation of Europe: hierarchical floristic classification system of vascular plant, bryophyte, lichen and algal communities. Applied Vegetation Science 19: 3‑264. https://doi.org/10.1111/avsc.12257; empty otherwise.

### Data set 3.

#### Data set name

EGVtable

#### Data format

xlsx

#### Data format version

csv

#### Description

Ecogeographical variables (as in Avotins et al. (2025)) that can be used as environmental drivers for each species when conducting habitat suitability modelling in Latvia.

**Data set 3.** 

Column label	Column description
egv_filename	File name of the standardised ecogeographical variable.
egv_layername	Layer name of the standardised ecogeographical variable.
egv_radius	Scale: '0' - 100 m, other values denote radius around the centre of 100 m cells.
longname_english	Long name of the ecogeographical variable in English.
longname_latvian	Long name of the ecogeographical variable in Latvian.
suggested_updating	Suggested layer updating frequency to reproject habitat suitability models.
egv_mean	Value used for centring.
egv_rms	Value used for scaling.
[CODE]	Ecogeographical variables selected for the species (columns 9:482 are named according to the species specific code used in this project).

### Data set 4.

#### Data set name

Observations

#### Data format

xlsx

#### Data format version

csv

#### Description

Observation file, containing expert-verified, spatially filtered observations for the species selected for habitat suitability modelling.

**Data set 4.** 

Column label	Column description
Group	Taxonomic group.
NR	Assigned taxon number within group.
ScientificName	Scientific name.
CODE	Species specific code used in this project.
LKS_X	X-coordinate of the observation, epsg:3059.
LKS_Y	Y-coordinate of the observation, epsg:3059.
Resolution	Spatial resolution (m) of the observation.
Year	Year of the observation.
Date	Date of the observation; empty if unknown.
Time	Time of the observation (24 hr clock, local timezone); empty if unknown.
LifeStage	Taxonomic group specific life stage; empty if unknown.
Status	Status of the observation; empty if unknown.
Sex	Sex - Male / Female; empty if unknown.
Abundance	Abundance.
Units	Abundance units; empty if unknown.
DataSource	Data source as recognised by experts: 'N.Surname' - expert personal data base; 'Agrihots' - Institute of Plant Protection Research "Agrihorts", Latvia University of Life Sciences and Technologies; 'Dabasdati' - Latvijas Dabas Fonds, Latvijas Ornitoloģijas biedrība (2024) Dabasdati.lv. https://dabasdati.lv/lv. Accessed on: 20-12-2024; 'GrassLIFE - project LIFE16NAT/LV/000262 (2017–2023) database (summaries available at https://grasslife.lv/wp-content/uploads/2023/03/D1Atskaite_LU_www.pdf and https://grasslife.lv/wp-content/uploads/2023/03/D4_Monitoring-report-demonstration-farm.pdf); 'GrassLIFE2' - project LIFE21-NAT-LV-GrassLIFE2 (2023-2028) database (not available publicly); 'LatViaNature' - project LIFE19IPE/LV/000010 database (species catalogue available at https://latvianature.daba.gov.lv/wp-content/uploads/2024/02/sugu-katalogs-WEB-LIFE-XS.pdf); 'LIFE for Species' - project LIFE19GIE/LV/000857 dataset (not available publicly); 'OZOLS' - Nature Conservation Agency (2025) Nature Data Management System OZOLS. https://www.daba.gov.lv/en/nature-data-management-system-ozols. Accessed on: 25-02-2025 (some original records published here might not be available publicly); 'PlutoF_observations' and 'PlutoF_specimens' - associated databases in portal PlutoF (Kõljalg U, Abarenkov K, Zirk A, Runnel V, Piirmann T, Pöhönen R, Ivanov F (2019) PlutoF: Biodiversity data management platform for the complete data lifecycle. Biodiversity Information Science and Standards 3: e37398. https://doi.org/10.3897/biss.3.37398).
DataSourceReliability	Relative reliability of data source. Three levels - citizen science (relatively low), expert/research (relatively high), mixed. Empty if unknown.
IDdatabase	Unique identificator as used in the source database; assigned "HiQBioDiv_[CODE]_[date]_[numeration]" in this project if unknown.
Mislocation_CLC	Indicator that observation is mislocated due to its CLC class.
Mislocation_Sea	Indicator that observation is mislocated due to location in sea.
AccumTCL_cell	Accumulated Tree Cover Loss (The Global Forest Watch v1.12 (Hansen MC, Potapov PV, Moore R, Hancher M, Turubanova SA, Tyukavina A, Thau D, Stehman SV, Goetz SJ, Loveland TR, Kommareddy A, Egorov A, Chini L, Justice CO, Townshend JRG (2013) High-Resolution Global Maps of 21st-Century Forest Cover Change. Science 342 (6160): 850‑853. https://doi.org/10.1126/science.1244693)) proportion at analysis cell since the year of observation, missing values in years prior to the baseline of this analysis.
AccumTCL_hr	Accumulated Tree Cover Loss (The Global Forest Watch v1.12 (Hansen MC, Potapov PV, Moore R, Hancher M, Turubanova SA, Tyukavina A, Thau D, Stehman SV, Goetz SJ, Loveland TR, Kommareddy A, Egorov A, Chini L, Justice CO, Townshend JRG (2013) High-Resolution Global Maps of 21st-Century Forest Cover Change. Science 342 (6160): 850‑853. https://doi.org/10.1126/science.1244693)) proportion at home range scale since the year of observation, missing values in years prior to the baseline of this analysis.
AccumDW_cell	Accumulated change of Dynamic World (Brown C, Brumby S, Guzder-Williams B, Birch T, Hyde SB, Mazzariello J, Czerwinski W, Pasquarella V, Haertel R, Ilyushchenko S, Schwehr K, Weisse M, Stolle F, Hanson C, Guinan O, Moore R, Tait A (2022) Dynamic World, Near real-time global 10 m land-use land-cover mapping. Scientific Data 9: 251. https://doi.org/10.1038/s41597-022-01307-4) class proportion at analysis cell since the year of observation, missing values in years prior to the baseline of this analysis.
AccumDW_hr	Accumulated change of Dynamic World (Brown C, Brumby S, Guzder-Williams B, Birch T, Hyde SB, Mazzariello J, Czerwinski W, Pasquarella V, Haertel R, Ilyushchenko S, Schwehr K, Weisse M, Stolle F, Hanson C, Guinan O, Moore R, Tait A (2022) Dynamic World, Near real-time global 10 m land use land-cover mapping. Scientific Data 9: 251. https://doi.org/10.1038/s41597-022-01307-4) class at home range scale since the year of observation, missing values in years prior to the baseline of this analysis.

### Data set 5.

#### Data set name

FilteringGuide

#### Data format

xlsx

#### Data format version

csv

#### Description

Observation mislocation filtering guide, containing annotations of land-cover classes that are unsuitable for each species.

**Data set 5.** 

Column label	Column description
Group	Taxonomic group.
NR	Assigned taxon number within group.
ScientificName	Scientific name.
CODE	Species specific code used in this project.
HomeRange	Species home range class (one of 500, 1250, 3000).
NeedsCLCcheck	Indicator to perform broad landscape class analysis for location errors, based on following fields (BuiltUp, OpenLandscape, Trees, Wetlands, Water, Sea); '1' - yes, '0' - no.
BuiltUp	Built-up class, CLC codes 111, 112, 121, 122, 123, 124, 131, 132, 133, 142, indicator '-1' denotes potential location error if observation is in this class.
OpenLandscape	Open landscape class, CLC codes 211, 222, 231, 242, 243, 321, 322, 331, indicator '-1' denotes potential location error if observation is in this class.
Trees	Tree covered class, CLC codes 141, 311, 312, 313, 324, 333, indicator '-1' denotes potential location error if observation is in this class.
Wetlands	Wetland class, CLC codes 411, 412, indicator '-1' denotes potential location error if observation is in this class.
Water	Water class, CLC codes 511, 512, 523, indicator '-1' denotes potential location error if observation is in this class.
Sea	Sea area, indicator '-1' denotes potential location error if observation is in this class.
StatusLULCchangeAnalysis	Indicator whether LULC change analysis has been conducted with three levels: '0' - not performed, '1' - partial analysis that should be repeated, '2' - detailed analysis.
EnvironmentLULCchangeAnalysis	Indicator what landscape classes should be analysed, four levels: ' 0' - not necessary, '1' - forests only, '2' - farmland only, '3' - all classes.

## Supplementary Material

D85B87F3-851C-52D1-AF21-748E770B4099 10.3897/BDJ.14.e194577.suppl1 Supplementary material 1 Summary of observation data sources Data type summary table Brief description This table provides the number of observations in the "Observations" dataset from each data source across species groups. File: oo_1711863.xlsx https://binary.pensoft.net/file/1711863 Rūta Starka

## Figures and Tables

**Table 1.** A summary of the number of taxa and their observations included in the dataset.

**Group**	**Number of nomenclature units**	**Number of taxa evaluated**	**Number of taxa selected for modelling**	**Number of observations**
Amphibia	14	14	11	6037
Araneae	2	2	2	76
Bryophyta	145	145	53	18209
Chiroptera	17	17	9	0*^1^
Coleoptera	993	993	42	5352
Diptera	6	6	6	434
Hymenoptera	19	19	18	1699
Lichen	658	658	12	1356
Mollusca	4	4	4	240
Orthoptera	44	44	11	849
Papilionoidea	108	108	24	7164
PoroidFungi	62	62	10	1172
Reptilia	8	8	6	2352
Tracheophyta	2135	2096	266	117246*^2^

## Footnotes

*1 Sharing Chiroptera observations are restricted (Nature Conservation Agency 2023);

*2 *Cypripedium calceolus* observations are excluded (Nature Conservation Agency 2023).
